# First assessment of POPs and cytochrome P450 expression in Cuvier’s beaked whales (*Ziphius cavirostris*) skin biopsies from the Mediterranean Sea

**DOI:** 10.1038/s41598-020-78962-3

**Published:** 2020-12-14

**Authors:** Matteo Baini, Cristina Panti, Maria Cristina Fossi, Paola Tepsich, Begoña Jiménez, Frazer Coomber, Alice Bartalini, Juan Muñoz-Arnanz, Aurelie Moulins, Massimiliano Rosso

**Affiliations:** 1 Department of Physical, Earth and Environmental Sciences, University of Siena, Via Mattioli, 4, 53100 Siena, Italy; 2 CIMA Research Foundation, Via Magliotto 2, 17100 Savona, Italy; 3 Department of Instrumental Analysis and Environmental Chemistry, Institute of Organic Chemistry (IQOG-CSIC), Juan de la Cierva 3, 28006 Madrid, Spain; 4 School of Life Sciences, University of Sussex, John Maynard Smith (JMS) Building, Falmer, Brighton, BN1 9QG UK; 5 The Mammal Society, 18 St, John’s Church Road, London, E9 6EJ UK

**Keywords:** Ecology, Environmental sciences

## Abstract

The Cuvier's beaked whale (*Ziphius cavirostris*) is one of the least known cetacean species worldwide. The decreasing population trend and associated threats has led to the IUCN categorising the Mediterranean subpopulation as Vulnerable on the Red List of Threatened Species. This study aimed to investigate for the first time the ecotoxicological status of Cuvier's beaked whale in the NW Mediterranean Sea. The study sampled around the 20% of the individuals belonging to the Ligurian subpopulation, collecting skin biopsies from free-ranging specimens. The levels of polychlorinated biphenyl (PCBs), polybrominated diphenyl ethers (PBDEs) and induction of cytochrome's P450 (CYP1A1 and CYP2B isoforms) were evaluated. Results highlighted that the pattern of concentration for the target contaminants was PCBs > PBDEs and the accumulation values were linked to age and sex, with adult males showing significantly higher levels than juvenile. Concerns raised by the fact that 80% of the individuals had PCB levels above the toxicity threshold for negative physiological effects in marine mammals. Therefore, these findings shed light on this silent and serious threat never assessed in the Mediterranean Cuvier’s beaked whale population, indicating that anthropogenic pressures, including chemical pollution, may represent menaces for the conservation of this species in the Mediterranean Sea.

## Introduction

Cuvier’s beaked whales (*Ziphius cavirostris*) are deep diving marine mammals^1^ with an almost sub-polar cosmopolitan global distribution^2^. However, due to its diving behaviour, subtle surfacing and typical offshore habitat, this species, like many other beaked whale species, it remains relatively understudied^3^. A global assessment has highlighted the existence of a genetically distinct subpopulation of Cuvier’s beaked whale in the Mediterranean Sea^4^. This subpopulation has been estimated at less than 10,000 mature individuals, with a speculated decreasing trend in abundance^5,6^. To date, three important geographical areas within the Mediterranean Sea have been identified for this species: Northern Ligurian Sea, Alboran Sea and Hellenic trench^5,7^.

Most research on Cuvier’s beaked whales in the Mediterranean has been driven by the need to assess the risks posed from noise pollution. One of the main threats to this species is exposure to mid- and low frequency underwater sound pollution^8,9^. This threat has been evidenced by multiple records of coincidental mass stranding events^6^, the known sensitivity of this species to military sonar^10^ and dive behavioural changes caused by passing ships^11^. Research on Cuvier’s beaked whales in the Mediterranean basin has been dedicated to reviewing stranding data^12^, characterising diving behaviour^1^, identifying habitat preferences^7,13,14^ and assessing density and abundance estimates^5^. All of which can be used to help identify the risk posed by anthropogenic noise pollution on spatial and behavioural scales.

The decreasing population trend and associated threats have led to the International Union for Conservation of Nature (IUCN) categorising this Mediterranean subpopulation of Cuvier’s beaked whale as Vulnerable on the Red List of Threatened Species^15^. It is, therefore, very urgent to investigate other potential stressors to define an effective risk assessment for the species, such as persistent organic pollutants.

Persistent organic pollutants (POPs) are chemical compounds of global concern due to their persistence in the environment, their ability to be transported over long distances^16^ and their effects on natural populations^17,18^. POPs are biologically active chemicals that have the ability to bioaccumulate and biomagnify within marine food webs^19^ and have the potential to negatively impact marine organisms^20^.

Given the impact and persistence of POPs on and within the environment, the use and manufacture of many of them have already been banned. Polychlorinated biphenyls (PCBs), man-made chlorinated organic chemicals, were banned globally in the 1970s^21^ and some polybrominated diphenyl ethers (PBDEs), a class of brominated flame retardants, started being banned around 20 years ago^22^.

In the Mediterranean Sea, the environmental concentrations of PCBs does not seem to show any noticeable decline^18,23^ and it is also reasonable for PBDEs to be present in the marine environment in large concentrations^22^. These chemicals are considered as legacy contaminants of re-emerging concern since it has been demonstrated to affect natural populations^17,18^.

Exposure to xenobiotic compounds, i.e. POPs, can cause immune system suppression^24^, and endocrine disruption^25,26^ in marine mammals. Concentrations of PCBs in different Mediterranean odontocete species have been reported surpassing toxicity thresholds for marine mammals^27^. Such concentrations can have population level consequences for marine mammals through reduced reproduction and/or survival^28,29^.

The induction of cytochrome P450 (CYP450) isoforms has been considered a biomarker of chemical exposure in marine mammals^30^. CYP1A1 and CYP2B have been detected in cetacean skin, and the induction of these isoforms has been related to the exposure to lipophilic contaminants (AhR inducers) such as organochlorine compounds, polycyclic aromatic hydrocarbons (PAHs), and PBDEs both ex vivo and in field studies^31–35^.

Since there have been no previous reports on the levels and potential impacts of persistent contaminants in Cuvier’s Beaked whale, this paper aims to investigate, for the first time, its ecotoxicological status in the Mediterranean Sea. Sampled animals inhabit the northern Ligurian Sea within the Pelagos Sanctuary, a Specially Protected Areas of Mediterranean Importance (SPAMI) located in the North-western part of the Mediterranean Sea. This is an important area for the species^7^ as well as an area subjected to high levels of anthropogenic impact^36–38^. This population is resident and also one of the most studied in the Mediterranean with long-term research starting in the late 1990s. Using photographic mark-recapture methods, the population size, estimated to be roughly 100 individuals^6^, and demographic situation is known for a large majority of these animals.

We analysed PCB and PBDE concentrations in blubber portion of biopsy samples collected. Besides, in order to assess the exposure to those anthropogenic contaminants, we evaluate the CYP isoforms (CYP1A1 and CYP2B) expression/induction in skin biopsies by Western Blot (WB) analysis. Contaminant accumulation and CYPs biological responses were further analysed considering demographic variables (i.e. sex and age) to elucidate the levels and the role of persistent contaminant accumulation and their potential effects on the resident Cuvier’s beaked whale population.

## Results

### Sample representativeness

**Figure 1:**
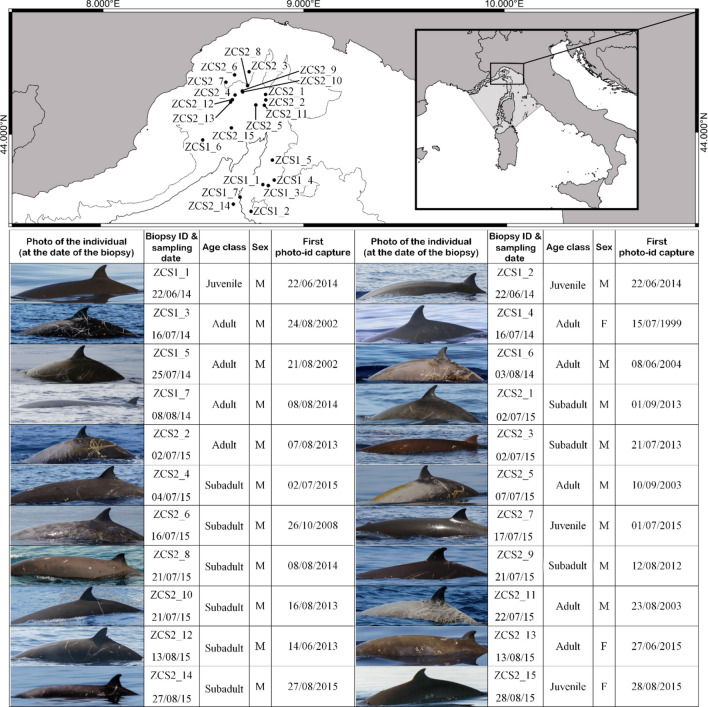
Map showing the study area, biopsy locations, the extent of the Pelagos Sanctuary, photos of sampled individuals, and information about sampling date, age class and their first photographic capture (Photos by M. Rosso). Map was generated using QGIS Geographic Information System (Version 3.10.10 'A Coruña'; Open Source Geospatial Foundation. http://qgis.org).

Samples were collected and analysed from 22 different individuals (Fig. 1). These represent 20–28% of the total resident population of Cuvier’s beaked whales in the study area (100 individuals with 95% CI = 79–112;^6^). Samples were successfully collected from individuals of three different age classes^39,40^: four juveniles (estimated age < 5-year-old), nine subadults (estimated age 5–10-year-old) and nine adults (estimated age > 10-year-old). Since only three females, one juvenile and two adults, were sampled, it was not possible to perform any statistical analysis between sexes. Therefore, the statistical analysis was focused on the differences between the three male age classes, of which the adult females were not included. The juvenile female (ZCS2_15) was included in the juvenile group, disregarding gender, due to the young age of the specimen (i.e. prepubertal individual). However, two samples, 1 subadult male and one adult male, did not containe enough blubber to analyse the concentrations of PCBs and PBDEs.

### Contaminants

All the 18 PCB congeners and 23 out of the 27 (85%) PBDE congeners were detected in every Cuvier’s beaked whale sample analysed. PCB concentrations were found to be higher than PBDEs and strong positive correlation was found between PCBs and PBDEs concentrations (rho = 0.97 *p* value < 0.001).

### PCBs

**Figure 2:**
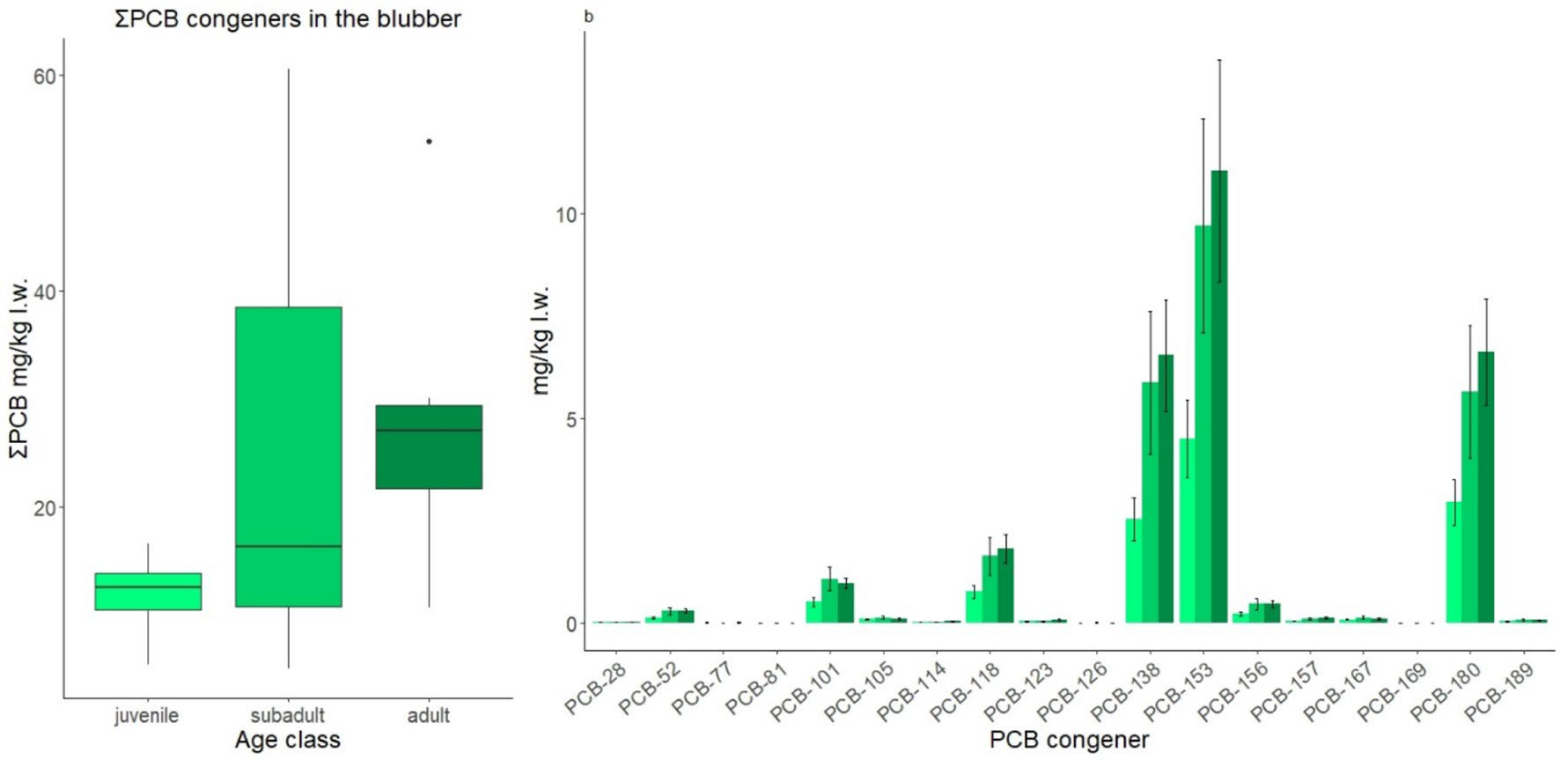
PCB levels in the blubber of Cuvier’s beaked whale in the three age classes: juvenile (light green), subadult (medium green) and adult (dark green). (**a**) Boxplots showing ΣPCB concentrations (mg/kg l.w.) in the blubber of juvenile (n = 4), subadult (n = 8) and adult males (n = 6). (**b**) The mean values of each individual PCB congener profiles for all 18 PCB congeners, in the age classes. Error bars represent standard errors (SE).

The median ΣPCB in males was found to be highest in adults (∑PCBs 27.13 mg/kg l.w.) then in subadults (∑PCBs 16.41 mg/kg l.w.) and lowest in juveniles (∑PCBs 12.41 mg/kg l.w.), with a significant difference between juveniles and adults (W = 21, *p* value = 0.033). However, no significant difference was found between the other groups (Fig. 2; Table 1). Although no statistical analysis was conducted between sexes in the adult group, the two adult females (ZCS1_4 = 3.06 mg/kg l.w. and ZCS2_13 = 8.22 mg/kg l.w., Supplementary Information 1, Table S1-1) displayed lower ∑PCBs levels, with a mean value (5.683 mg/kg l.w.) much lower than that of adult males (28.165 mg/kg l.w.: Table 1 and Supplementary Information 1, Table S1-2).

**Table 1:** Mean, median, standard deviation of ΣPCB, ΣICES7, and ∑PBDE, in the blubber of Cuvier’s beaked whale of juvenile (n = 4), subadult male (n = 8), adult male (n = 6) concentrations are expressed in mg/kg lipid weight.

	Juvenile			Subadult			Adult male			Adult female		
	Mean	Median	SD	Mean	Median	SD	Mean	Median	SD	Mean	Median	SD
∑PCBs	11.82	12.58	4.68	25.11	16.41	19.91	28.17	27.13	14.40	5.64	5.64	3.65
ΣICES7	8.82	9.43	3.44	18.34	12.29	14.24	20.73	19.37	10.77	4.08	4.08	2.38
∑PBDEs	0.26	0.27	0.11	0.62	0.35	0.60	0.49	0.50	0.15	0.14	0.14	0.13
Total TEQ	102.48	110.89	44.06	242.59	129.15	305.37	157.44	167.51	28.04	162.39	162.39	178.56
BDE-47/PCB-153	0.03	0.03	0.01	0.03	0.03	0.01	0.02	0.03	0.01	0.03	0.03	0.02
BDE-47/BDE-99	2.36	2.59	0.52	2.60	2.63	0.14	2.62	2.58	0.16	2.22	2.22	1.00
BDE-99/BDE-100	1.37	1.31	0.17	1.30	1.28	0.15	1.57	1.55	0.20	1.49	1.49	0.19
BDE-153/BDE-154	0.38	0.37	0.05	0.36	0.35	0.04	0.45	0.45	0.06	0.40	0.40	0.11

Total TEQ is expressed in pg/g lipid weight. Mean, median and standard deviation, of BDE-47/PCB-153, BDE-47/BDE-99, BDE-99/BDE-100, BDE-153/BDE-154 ratio.

Abundance of PCB congeners in the blubber were PCBs 153 > 180 > 138 > 118 > 101, which together contributed to the majority (95%) of the ΣPCB content in Cuvier’s beaked whale samples (Fig. 2). The highly persistent PCB-153 congener represented the main PCB compound, accounting for 38%, 38.6% and 39% of the ΣPCB concentration in juveniles, subadults and adults, respectively. The individual PCB congener profiles also displayed similar patterns to the ΣPCB profile between the age groups with adults generally having higher concentrations than sub-adults and juveniles (Fig. 2).

To further investigate the PCB results, each congener was assigned to a group. The first groups were based on the degree of chlorination (TriCB, TetraCB, PentaCB, HexaCB and HeptaCB). Then there were the non-*ortho*-dl-PCBs and mono-*ortho*-dl-PCBs. Four Structure Activity Groups (SAGs 1–4) were assigned based on their persistency and capacity for biotransformation as previously defined^41^ (Supplementary Information 1). One group was based on the PCB congeners listed by the International Council for the Exploration of the Sea (ICES: Table 1 and Supplementary information 1), here on referred to as ∑ICES7.

In all groups, except for TriCB and non-*ortho* PCBs the mean concentration values were highest in adults and lowest in juveniles (Supplementary Information 1), indicating that these PCB group concentrations increased with age. However, due to the high variation among individuals these differences were not statistically significant.

### PBDEs

Unlike PCBs not all PBDEs were identified in all the sampled animals. Four PBDE congeners (3, 7, 15, 126) out of the 27 analysed were consistently not detected in any sample.

**Figure 3:**
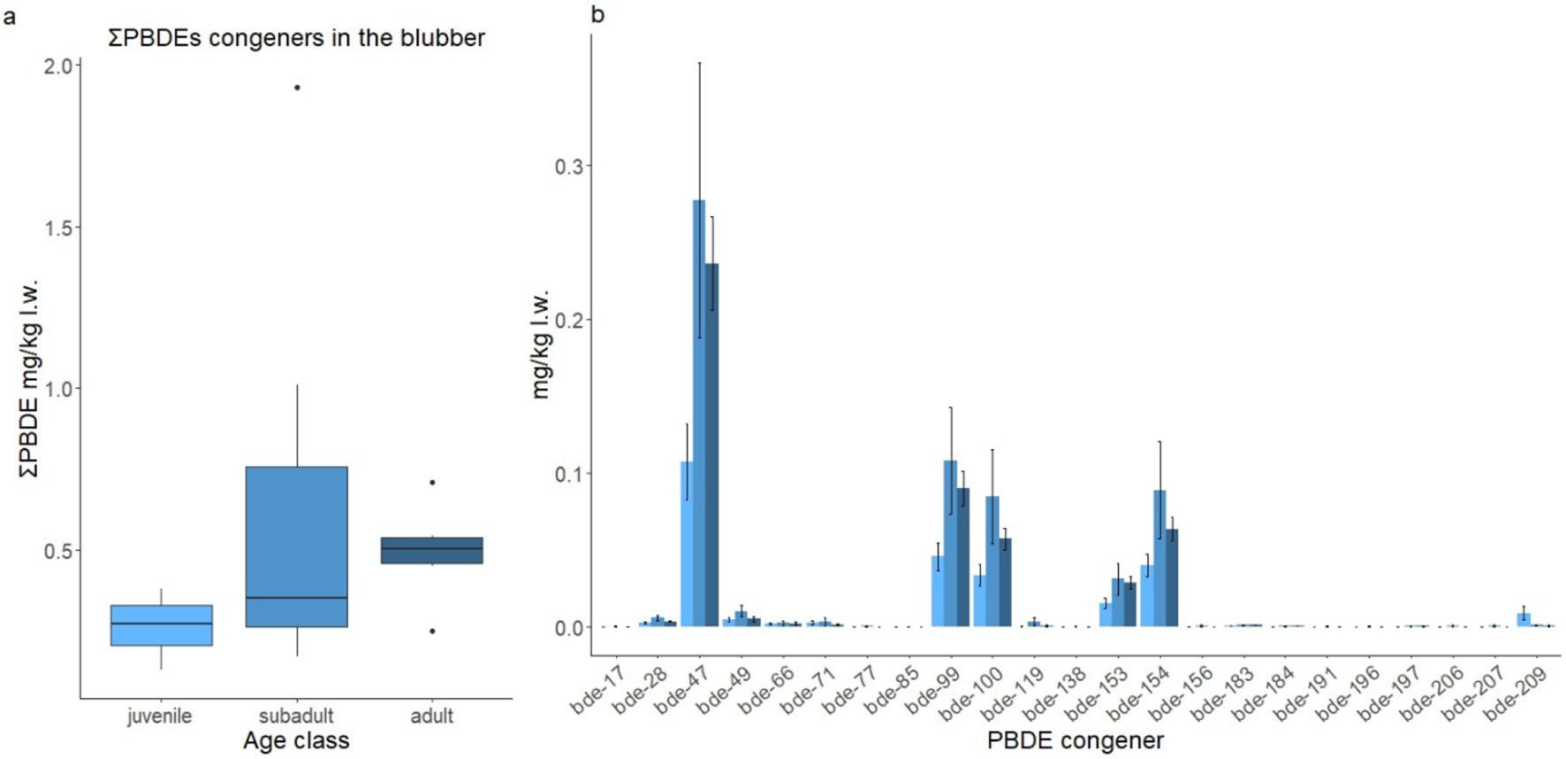
PBDE levels in the blubber of Cuvier’s beaked whale in the three age classes: juvenile (light blue), subadult (medium blue) and adult (dark blue). (**a**) Boxplots showing ΣPBDE concentrations (mg/kg lipid weight) in blubber of Cuvier’s beaked whale of juvenile (n = 4), subadult male (n = 8) and adult male (n = 6). (**b**) The mean values of the individual PBDE congener for all 27 PBDE congeners, in the age classes. Error bars represent standard errors (SE).

Regarding the concentrations of ΣPBDE found in the different age groups, the trend was similar to those observed for PCBs. The median value in adults (ΣPBDE = 0.503 mg/kg l.w.) was higher than in subadults (ΣPBDE = 0.351 mg/kg l.w.) and juveniles (ΣPBDE = 0.271 mg/kg l.w.). ΣPBDE were significantly higher in adults than in juveniles (ΣPBDE W = 22, *p* value  = 0.01905), while no difference was found between the other groups (Fig. 3). As highlighted for PCBs, the two adult females showed PBDEs values lower (ZCS1_4 = 0.054 mg/kg l.w.; ZCS2_13 = 0.231 mg/kg l.w.) than those shown in adult males. The PBDEs congener profile shown in Fig. 3, was dominated by lower-medium brominated congeners. The most abundant PBDE congeners were BDE 47, 99, 154, 100, 153 whose average contribution to the total PBDE content was 45%, 17%, 14%, 13%, and 5%, respectively.

The different PBDEs were then grouped in the respective commercial mixtures Deca-, Octa- and Penta-BDE^42^ to compare profiles between the age groups. The results show that penta-BDE is the most dominant component among all age classes, followed by Octa-BDE mixture and Deca-BDE mixture. It is interesting to note that the concentrations of the deca-BDE mixture decrease as the age of the whales increases. Despite the correspondence between PBDE congeners found in blubber of Cuvier’s beaked whale and main components in the technical mixtures, the relative abundance of these compounds differed from that found in the commercial formulations^42^.

### Metabolic biotransformation of PCBs and PBDEs

To investigate the metabolic biotransformation capacity of Cuvier’s beaked whale for the investigated compounds, the ratio between the most dominant compounds (BDE-47 and PCB-153) was calculated^43^. Results showed that BDE-47/PCB-153 ratios were below 1 in all age classes. The highest values were observed in subadults followed by juveniles and adults (Table 1). BDE-47/PCB-153 ratio did not differ statistically among age classes (subadult vs. adults: W = 31, *p* value = 0.4136; subadult vs juveniles: W = 20, *p* value = 0.5697; adult vs juveniles: W = 11, *p* value = 0.9143).

Regarding PBDE congeners, BDE-99/100, 153/154 and 47/99 ratios have been used to assess possible differences in metabolic capacities of these classes of compounds as well as bioaccumulation patterns^44,45^, which may vary among age classes.

BDE-99/100 and BDE-153/154 ratios had similar trends with the highest value in adults (mean value: 1.57 and 0.45, respectively) followed by juveniles (mean value: 1.37; 0.38) and subadults (mean value: 1.30; 0.36). BDE-99/ BDE-100 ratio was significantly higher in adults than subadults (W = 5, *p* value = 0.006) and then juveniles (W = 21, *p* value = 0.033). Concerning BDE-153/ BDE-154 ratio, results were significantly higher in adults than subadults (W = 42, *p* value = 0.010), while no other differences were found among the other groups. BDE-47/99 ratios in the three age classes were higher in adults (mean value: 2.62) and subadults (mean value: 2.60) than in juveniles (mean value: 2.36).

### Toxicity assessment

**Figure 4:**
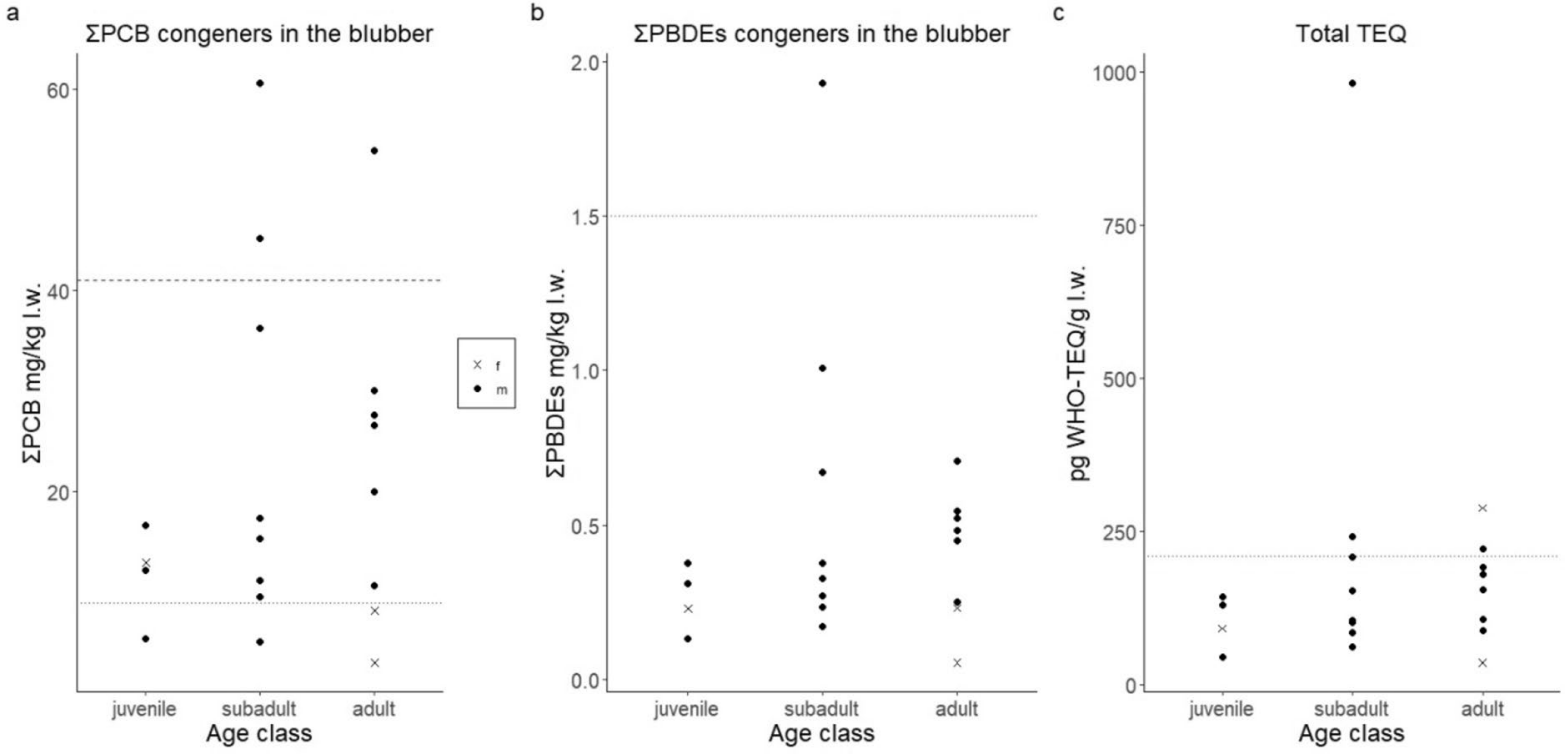
Contaminant concentrations for juvenile (n = 4), subadult male (n = 8), adult male (n = 6) and adult females (n = 2), in relation to ΣPCB, ΣPBDE and TEQs published toxicity threshold. (**a**) ΣPCB (mg/kg l.w.) concentrations in relation to published toxicity thresholds. The lower dotted line represents the lower toxicity threshold (9 mg/kg l.w.) published for marine mammals^18,47^. The dashed line represents the highest threshold (41 mg/kg l.w.) published for marine mammals^48^. (**b**) ΣPBDE (mg/kg l.w.) concentrations in relation to 1.5 mg/kg l.w. published toxicity threshold^49^. (**c**) TEQ concentrations (pg WHO-TEQ/g l.w.) in relation to 210 pg WHO-TEQ/g l.w. published toxicity threshold^50^.

In order to assess the potential toxicological effect of the contaminants analysed, they were evaluated in relation to the threshold limits proposed in the literature. Two PCB toxicity thresholds were used in this study as proposed previously in other toxicological studies on cetaceans^17,18,46^. The lowest value (9.0 mg/kg l.w. ΣPCB) was proposed as the toxicity threshold concentration for the onset of physiologic effects in marine mammals^18,47^. The highest value (41 mg/kg l.w. ΣPCB) was proposed as a toxicity threshold for reproductive impairment in Baltic ringed seals (*Pusa hispida*^48^). The ΣPCB values detected in most of our sampled individuals (80%) exceeded the lower limit, and three of them showed ΣPCB values above the highest threshold limit (two subadults and one adult: Fig. 4a).

Regarding ΣPBDE, we set the threshold at 1.5 mg/kg l.w., which has been found to be the value associated with endocrine disruption in grey seals^49^. ΣPBDE concentrations detected in the different whales belonging to the three age class are all below the threshold limit except for one subadult specimen (Fig. 4b).

The potential toxicity of dl-PCBs is commonly assessed by the Toxic Equivalent Quantity (TEQ) approach, in which each's congener toxicity is relativized to that of the most toxic one, the 2,3,7,8-TCDD^51^. Calculated TEQs ranged from 44 to 982 pg WHO-TEQ/g l.w. It should be highlighted that in the blubber of two adults (ZCS1_3 and ZCS1_13) and two subadults (ZCS2_8 and ZCS2_9) analysed exceed the threshold of 210 pg WHO-TEQ/g l.w. the proposed threshold of immunosuppression in harbour seals^50^ (Fig. 4c). However, it is important to stress that there is a wide inter-species sensitivity towards different contaminants and that there are no specific studies on this species, and few on other marine mammals.

### Cytochrome P450

**Figure 5:**
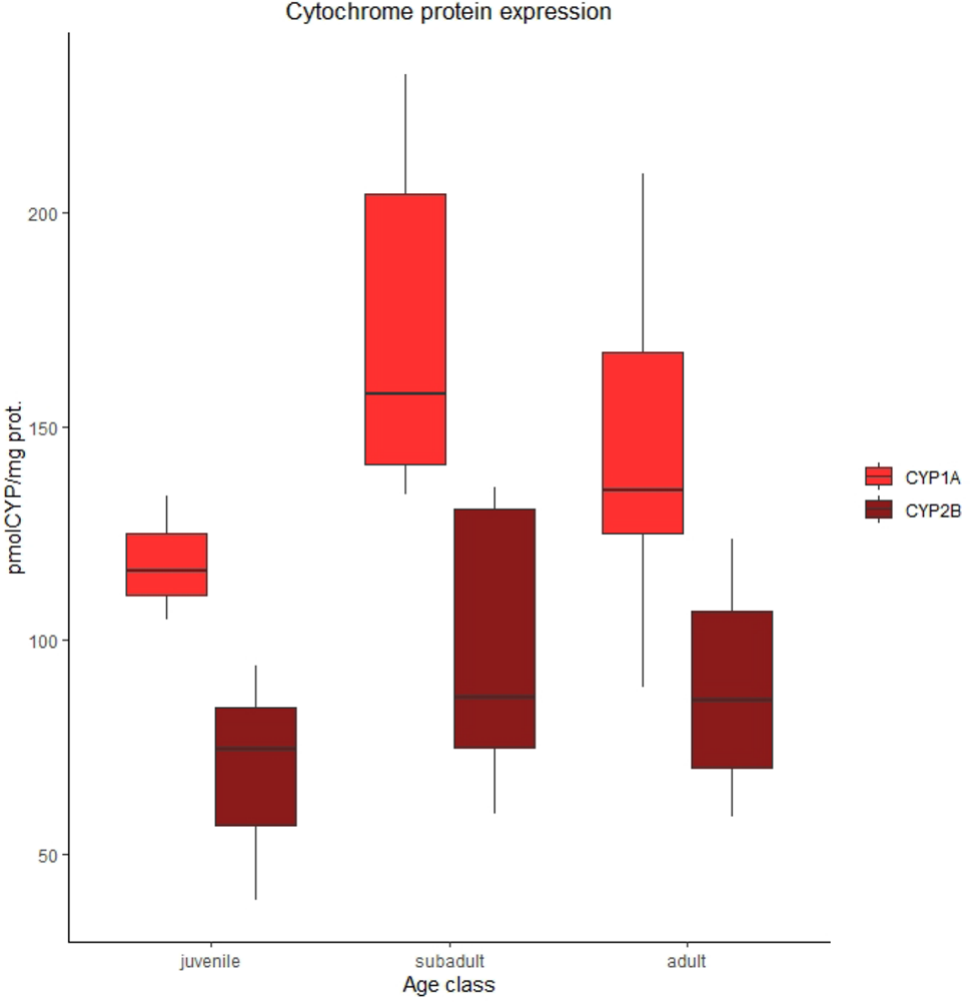
Boxplots showing CYP1A1 and CYP2B expression (pmolCYP/mg protein) in juvenile (n = 4), subadult male (n = 8) and adult male (n = 6). Error bars represent standard errors (SE).

The detection of cytochrome CYP450 expression was carried out for the first time on this species. This analysis was successfully accomplished using the Western Blot technique which allowed the identification and quantification of cytochrome-like bands for the CYP1A1 (59 kDa) and CYP2B (56 kDa) in the dermal part of skin biopsies. The protein expression in different age classes was similar for cytochromes 1A1 and 2B with the highest values in subadults followed by adults and juveniles (Fig. 5). CYP1A1 protein expression differed significantly between juvenile and subadult, with subadults having a higher expression (W = 27; *p* value = 0.004), while no differences were noted for CYP2B. Adult females showed CYP1A1 and CYP2B expression more similar to juveniles than adult males.

A significant positive correlation was found also between CYP1A1 and CYP2B concentrations (rho = 0.59, *p* = 0.009). In order to evaluate the influence of PCBs and PBDEs on the expression of CYP1A1 and/or CYP2B, we ran a regression analysis on CYP1A1/CYP2B concentration against single PCB congeners, ΣPCB, different PCB groups (TriCB, TetraCB, PentaCB, HexaCB and Hepta CB, non-*ortho*-dl-PCBs, mono-*ortho*-dl-PCBs and four SAGs), single PBDE congeners, ΣPBDE and Deca-, Octa- and Penta-BDE mixtures. A check for collinearity was conducted to assess the correlation between the PCB groups. Variables showing correlation > 0.8 were not included in the regression analysis. Regression analysis was run using only ΣPCB, TriCB and non-*ortho* PCB, but none of these components showed a statistically significant correlation with CYP1A1/CYP2B. No correlation was found between CYP1A1 and PBDEs. Similarly, no correlation was found when looking at CYP2B and PBDEs concentrations.

## Discussion

This represents the first ecotoxicological study on free-ranging Cuvier's beaked whales both in the Mediterranean Sea and worldwide. Sample size (more than 20% of the estimated resident population in the Ligurian Sea) is unique for this kind of study and, above all, for this elusive species. Considering the amount of collected and analysed samples, and also the evenly distribution among age classes, the results may be extrapolated to the entire Cuvier’s beaked whale population in the area.

An added value of this study is the analysis of free-ranging individuals from a well-known population, which have been continuously studied (through mark-recapture) in the last twenty-years (since the 1990s). The mark recapture data previously collected allowed an analysis of contaminants concentrations and CYPs expression according to age and life-history of most of the individual sampled and to further provide new insights on the population status.

The analysis of levels and congener profiles of the two classes of contaminants investigated in this study could also provide information about possible biological and environmental differences among age groups. The variation in congener profiles among species and age classes is likely to be driven by several factors such as differences in prey preferences during the different life stages, differences in the physiochemical, physiological and metabolic processes that occur between females and males and age classes from juveniles to adults^25,52^.

Currently, few studies on contamination on beaked whales were published. Only three papers specifically report the PCBs and/or PBDEs levels in stranded Cuvier’s beaked whales outside the Mediterranean Sea^53–55^.

The pollutant load is also reported for other three species of beaked whales that feed at the same trophic level from Japanese waters (*Berardius bairdii*^56,57^, *Mesoplodon stejnegeri*^58,59^ and *Mesoplodon carlhubbsi*^60^) and from Canadian waters (*Hyperoodon ampullatus*)^34^.

Contaminant concentrations found in our study are higher than those reported in previous research for beaked whales, confirming that the Mediterranean Sea represents a contamination hotspot for marine mammals^27,52^.

Comparing the levels of ∑PCBs and ∑PBDEs with those present in the literature for the other cetacean species living in the same area, it can be highlighted that Cuvier’s beaked whale showed higher levels than fin whale did^52,61,62^. Among the odontocetes the values are lower to those reported for striped dolphin^31,37,63^, common bottlenose dolphin^52^, Risso’s dolphin^64^, pilot whale^62,64^ and similar to those observed in the sperm whale^62,65^. Differences observed were expected because of trophic level differences among the species, different prey preferences and both feeding habits and feeding ground areas.

In our study a strong positive correlation was found between PCBs and PBDEs concentrations suggesting a similar bioaccumulation of these substances in Cuvier’s beaked whales and a possible common source of contamination through their diet, mainly composed by mesopelagic squid^2,6^. The few published data on this species referred to individual specimens^53,54^, which does not allow to investigate how sex and age variables affect the accumulation of ∑PCBs and ∑PBDEs in this species. The increase of contaminant concentrations in blubber through the different age classes observed in the male whales, confirms what was previously described in harbour porpoises^66^, bottlenose dolphins^29^ and killer whales^67,68^. Therefore, while contaminant concentrations in males either remain stable or increase with age, in sexually mature females contaminant burdens generally decline. This may be due to the transfer of organic pollutants from females to offspring through gestation and lactation^46,69–73^. This was also observed in Cuvier’s beaked whale where the ∑PCBs and ∑PBDEs levels of the two adult females examined were lower than those of the adult males and similar to those of the young specimens. Thanks to the long-term studies conducted on this population we know that one of the females (ZCS1_4), has given birth to at least 4 calves since 1999 to the sampling date. This whale was, indeed, the one that had the lowest levels of ∑PCBs and ∑PBDEs in the dataset.

Among PCBs, the PCB-153 was the predominant congener in all age groups followed by PCB-180 and 138. This congener distribution and abundance also reflects the high deep-sea sediment and organisms’ affinity towards higher chlorinated congeners relative to that of low chlorinated compounds, suggesting the ease for the former to be transported from surface waters to the deep-sea^44^. However, the same trend was also observed in other beaked whales^34,60^ and marine mammals in general^46,62,74–76^, since these compounds are non biotransformable in cetaceans^41,77,78^. The abundance profile of non-*ortho*-dl-PCBs in the study samples (77 ≈ 126 > 169 > 81) is similar to other beaked whales^56,57^ but is remarkably different from that reported in blubber from Mediterranean sperm whales (126 > 169 > 77 > 81^65^). This is interesting given that diet is the main route of POP uptake for marine mammals and both species are deep divers sharing common prey (i.e. mainly mesopelagic squids)^6,64^. However, it is known that many factors such as lipid content, protein-binding, blood transport, or chlorination level, among others, affect the distribution and partitioning of PCBs and other POPs across tissues in marine and terrestrial species^79^. Thus, different toxicokinetics are likely to be considered behind the dissimilar abundance of non-*ortho* congeners in the blubber of these Mediterranean species instead of PCB concentration differences in their feeding habit and grounds.

The most abundant PBDEs were lower-medium brominated congeners BDE-47, BDE-99 and BDE-100, that represent the main components in the commercial penta-BDE formulations^42^. The results of this study were consistent with previous studies that reported a decrease in bioaccumulation with an increase in bromination^62,65,80,81^ as observed in other beaked whales^58^ and in aquatic food webs in general^82^. Although metabolism plays an important role in the PBDE congener accumulation in aquatic organisms, lower brominated congeners have higher biomagnification potential and, thus, possible toxicological effects^44,80^.

BDE-154 levels were also significant in all age classes. BDE-154 has been suggested to be a debromination product of BDE-183, the main congener in the technical octa-BDE mixtures^83,84^.

An interesting result was the increase of higher brominated BDEs in younger Cuvier’s beaked whales (juvenile > subadult > adult). This evidence needs to be further investigated but a possible explanation could rely on the prohibitions of Penta- and Octa-BDE commercial mixtures since 2004 (Directive 2003/11/EC of the European Parliament and of the Council). Juvenile individuals have therefore never come into direct contact with these commercial mixtures (unless through lactation), and have a higher percentage of BDE-209, which constitutes between 92 and 97% of the total BDE content in the deca-BDE formulations^42^. Possible metabolic differences in biotransformation of PCBs and PBDEs were investigated by calculating the ratios of the most dominant compounds, BDE-47/PCB-153. The lowest ratios were found in the adults, which indicates that the juveniles and subadults have a lesser developed ability for metabolic biotransformation of BDE-47, confirming what was also described in harbour porpoises from the North Sea^43^. With regard only to PBDE metabolism, the results of BDE-99/100, 153/154 and 47/99 ratios suggest that subadults could have greater metabolic capacities of this class of compounds. Moreover, BDE congener ratios could also reflect some differences in the feeding strategies among age classes, as shown for other marine species^44^. However, studies on the feeding ecology of this species in the study area are required to confirm this hypothesis.

The two CYP isoforms protein expression has been evaluated in Cuvier’s beaked whale in the present study for the first time. The qualitative and quantitative CYP1A1 and 2B protein analysis showed a correlation between the two isoforms and a significant difference between age classes with the highest levels of expression in subadult males compared to juvenile and adults. A similar trend has been found, for instance, in pilot whales from North-eastern Atlantic^85^ and false killer whales from Hawaii^86^. It is worth noting that the levels of CYP1A1 and 2B protein induction in the two adult females are among the lowest of the dataset. This can be related both to the influence of estrogen concentrations on CYP expression due to cross-talk between the Aryl hydrocarbon Receptor (AhR) and estrogen receptor (ER) and the subsequent activation or impairment of estrogen responsive gene expression in the presence of lipophilic compounds^87^ and both to the lowest levels of PCB and PBDEs accumulation in adult females due to reproductive and physiological status. However, the regulation of CYP expression patterns in Cuvier’s beaked whales may be affected by and be dependent on complex biological and metabolic pathways. These are related to the diet and physiology of this species, which are poorly investigated and need further investigation. No correlation was found between the two classes of compounds detected in the blubber (including any category shown in Table 1) and CYP1A1 and CYP2B analysed in the skin of Cuvier’s beaked whale. Also focusing on correlating specific compound which induce specifically the two isoforms, planar compounds (non- and mono-*ortho* PCBs) and CYP1A1 expression, and non-planar compounds (> 2 *ortho* chlorine compounds) with CYP2B, any statistical correlation exists among all age classes. Our data show that the PCBs and PBDEs, which accumulated in the blubber (at least in the outer part of the blubber sampled by skin biopsy remote dart sampling), did not correlate with the induction of CYP isoforms expression. Comparing our data to the only other beaked whale species analysed, *Hyperoodon ampullatus,* was also found to have no correlation between CYP1A1 expression and detected contaminants^34^. However, despite this is true also in some other cetacean species, including deep-divers^33,86^, it should be considered that older animals accumulate higher POP levels due to biomagnification but CYPs expression change in the context of maturation and aging being less efficient than younger animals^88^ and posing a further risk to the adult males. An additional analysis on the CYP isoforms was performed correlating SAGs with the protein expression. SAG 1, 2 and 5 are considered highly persistent because of their high level of chlorination and, therefore, are poorly metabolized, whereas SAG 3 and 4 are less persistent because they are less chlorinated and more easily metabolized by CYP1A1-mediated enzymes. Thus, this classification appears more appropriate to assess the potential ecotoxicological effects on cetaceans exposed to PCBs. The SAG classification approach became necessary since cetaceans have reduced capability of metabolising non dl-PCBs; therefore, these compounds can accumulate and become toxic to the organisms because of their low CYP2B enzyme activity, compared to other mammal species^41^. Moreover, a wide range of toxic effects were reported for non dl PCBs, which can act through a non-AHR-dependent response^89^. In this study no significant correlations were found between specific SAG congeners (1–4) and CYP1A1 and CYP2B protein expression in all three age classes. This suggests that the protein induction could be due to exposure to other classes of contaminants rather than those measured. It could also be that the levels of exposure that cause effects and negative physiological responses (e.g. age-related CYPs expression) in this species can be different from other cetacean species. Considering also the toxicity assessment of the compounds investigated, any significant relationship between calculated TEQ and CYP1A1 and CYP2B expression were shown. This evidence, already observed in other studies^86^, suggests that CYP1A1 and 2B expression can be related to broader and different AhR agonists not measured in the present study.

To gain a better understanding of the significance of the PCB and PBDE findings, toxicity profiles need to be quantified. This is a difficult task due to the vast range of mechanisms, toxicities, and synergistic and antagonistic interactions inter-species sensitivity. This is further confounded by the absence of specific studies on this species and the limited number of similar studies on other marine mammals. The use of Toxic Equivalency Factors (TEFs) to achieve TEQ constitutes an accurate approach for toxic risk assessments associated with dioxin-like compounds. This permits cetacean based evaluations on the different contribution of each pollutant to the TEQ^51^.

Overall, in our study only four whales (20% of dataset) exceed the threshold of 210 pg WHO-TEQ/g l.w. in blubber, proposed as the starting point for immunosuppression in harbour seals^50^. TEQ values are the lowest amongst those tested for other toothed whales in the Mediterranean Sea^62,65^, but this could be due to lack of PCDD/Fs contribution in our study.

However, over 80% of the whales considered in this study exceeded the lower toxicity threshold of 9 mg/kg l.w. for PCBs, the level at which marine mammals suffer physiological effects^47^. Furthermore, three animals (15%) exceeded the higher PCB threshold of 41 mg/kg l.w.^48^. The percentage of animals surpassing these thresholds is lower than what has been observed in striped^18^ and the bottlenose dolphins^46^. As discussed by several scientific papers^18,25^, the lower toxicity threshold may overestimate the true PCB risk to cetaceans, however, it is important to stress that there is a wide inter-species sensitivity towards different contaminants and that there are no specific studies on this species, and few on marine mammals^47^.

The risk posed to Cuvier’s beaked whales from PBDEs may be low as all whales, in all age classes, were below the toxicity threshold limit, unlike other cetacean species in the Mediterranean^52^ or elsewhere in the world^80^.

However, it is important to understand that the responses to the toxic effects of contaminants is highly specific to individual’s physiological status, nutrition state, body size and age. Therefore, caution is needed in applying such threshold levels on large cetaceans^47,70^ failure to do so may lead to an over or underestimation of the risk posed to a species or population.

All these data suggest that the health of a population, exposed to a mixture of known and unknown compounds, may depend not only on the levels of contaminants accumulated, but also on the effects which may occur when the levels of accumulation of environmental contaminants are below or near the established threshold levels.

For all these reasons, the integration of the data on the population structure and dynamics and the data on PCBs, PBDEs and CYPs responses on a poorly studied species and genetically isolated population, in an area of high ecological value and, also, taking into consideration the life history of each individual, makes the study unique for living cetaceans in the Mediterranean Sea.

All whales sampled were found to have detectable levels of PCBs, so at a minimum 20% of the population is affected and it could be assumed that all Cuvier’s beaked whales in the Pelagos Sanctuary have some levels of POPs. Furthermore, given that 80% were well above toxicity thresholds, it could be said that a large proportion of population could undergo negative physiological effects (including endocrine disruption).

These findings shed light on an additional anthropogenic threat, the chemical pollution, poorly considered so far for Mediterranean Cuvier’s beaked whales and its conservation, that should be considered—together with the anthropogenic noise—the main threat for this species in the Mediterranean sea^12^. Characterizing the exposure and effects to a plethora of threats in wild cetaceans can pave the way towards the comprehension of the actions needed for the effective conservation of specific populations and, ultimately, species.

## Methods

### Permits, ethics statement and approval

Biopsies were obtained in accordance with the relevant guidelines and regulations imposed by the Ministry for Environment, Territory and Sea (MATTM) and under sampling permits n. 0018799/PNM released from the same Italian institute (Div. II) and the Italian National Institute for Environmental Protection and Research (ISPRA). The research permits also included the necessary ethical approval in terms of sample collection, analysis and use for scientific studies.

### Biopsy collection

Biopsies of Cuvier’s beaked whales were collected in the Ligurian Sea (NW Mediterranean Sea) in 2014 and 2015. A 60-foot sailing vessel was used as a research platform allowing the research crew to reach the core of the study area and stay at sea overnight during consecutive days. The vessel towed a 4.3 m long 20 hp RHIB that was used to approach the whales during biopsy collection operations. Skin biopsies were collected in a specific dorso-lateral area below the dorsal fin using a 68 kg draw weight recurve crossbow (Barnett Panzer V). We obtained about 1 g of tissue samples using stainless steel biopsy tips (30 mm × 8 mm) attached to 18′' bolts. Tips were sterilized before use. The tissue samples obtained were stored in liquid nitrogen a few minutes after biopsy collection and stored at − 80 °C until laboratory analysis.

### Age class classification

All the whales biopsied have been sized in situ and photographed in order to allow both age classification and individual identification (i.e. only individuals showing adequate natural marking have been identified). All the whales photographed were separated into three different age classes—juveniles, subadults, adults. Animals were categorized in the age classes by both the estimated size and coloration patterns^39,40^. All the subadult and adult whales biopsied were well-marked allowing for individual identification. The photographs of the individuals identified were compared with each other and to photographs from 158 previously identified individuals collected during previous years (since 1998) in the same study area (CIMA Foundation database) gaining information on the life history of the sampled animals and making a crude estimation of the possible age.

### Sex determination

The sex was first estimated in the field based on size, coloration pattern, natural marking severity and individual history if the animals were already present in the photo-id catalogue. Sex was then confirmed by molecular sex determination based on ZFY/ZFX gene^90^. DNA was isolated by 20 mg of dermal tissue homogenised with a Tissue Lyser (Qiagen). The Wizard SV Genomic DNA Purification Kit (Promega) was used for DNA isolation according to the manufacturer's instructions. DNA was quantified by Nano-Drop ND-100 UV–Vis spectrophotometer (NanoDrop Technologies LLC) and purity assessed by 280/260 nm and 260/230 nm ratios. Gender was determined using 50–100 ng of DNA in standard PCR reactions following the protocol described by^90^.

### Cytochrome analysis: Western Blot

Analysis of cytochrome P450 CYP1A1 and CYP2B, used in this study as marker of POPs exposure, were analysed in the dermal part of the skin biopsies of Cuvier’s beaked whale using western-blotting (WB) techniques followed by a semi-quantitative analysis of the data^91^. A triplicate skin standard for odontocetes was used as internal standard during all the WB procedures to monitor the accuracy of the analytical method.

Sub-samples of biopsies (about 30 mg) were weighted and homogenized using a Tissue Lyser (Qiagen) in aryl-hydrocarbon-receptor (AhR) buffer (1:10)^92^. The homogenates were centrifuged and supernatants (S9 fractions) were collected and immediately frozen at -80 °C until analysis. Proteins were separated by 10% polyacrylamide gels (SDS-PAGE, Criterion XT Precast Gel, Bio-Rad) and blotted onto nitrocellulose sheets for 1 h at 375 mA. The membranes were saturated with a blocking solution for 1 h at room temperature and then incubated over-night with primary polyclonal anti-bodies Goat anti-rabbit CYP1A1 and anti-CYP2B (Oxford Biochemical Research; Oxford MI, USA), diluted 1:5000 and 1:1000 respectively, in TTBS 1% gelatin. Incubation with secondary antibody anti-goat HRP-labelled (Immun-Star-HRP-Chemiluminescent-Ki, Bio-Rad), diluted 1:3000 in TTBS 1% gelatin, was performed for 90 min at room temperature and protein detection was carried out according to the manufacturer’s instruction. Semi-quantitative analysis was performed using the Quantity One software (1-D Analysis Software, Bio-Rad). Molecular weights for CYP1A1 and CYP2B peptide have been calculated by the lane-based functions with multiple regression models using as a Precision Plus Protein Standard (Bio-Rad).

### Contaminant analysis

#### Sample processing

PCBs and PBDEs were analysed in blubber samples using the method described in^65^. Briefly, pollutants were extracted in a Soxhlet apparatus (24 h) with a n-hexane: dichloromethane (9:1) mixture. Samples were previously homogenised with anhydrous sodium sulphate (Na_2_SO_4_) and spiked with a suite of ^13^C-labeled standards of target contaminants. Resulting extracts were purified by means of the automated sample preparation system DEXTech + (LCTech GmbH, Dorfen, Germany) providing two fractions: F1 containing PBDEs and PCBs (save non-*ortho* PCBs), and F2 containing non-*ortho* PCBs. Fractions were evaporated using a TurboVap (Zymarck Inc., Hopkinton, MA, USA) system until ~ 1 mL and dried under a gentle nitrogen stream. A few microliters of ^13^C-labeled injection standards of PCBs and PBDEs were used for reconstitution of each sample prior to instrumental analysis. The lipid content of samples was obtained gravimetrically from weight measures before and after extraction. Comprehensive details on the whole sample analysis can be found in the Supplementary Information 2 (SI).

#### Instrumental determination

Six indicator PCB (PCB-28, 52, 101, 138, 153, 180), twelve dl-PCBs (PCB-77, 81, 105, 114, 118, 123, 126, 156, 157, 167, 169, 189) and twenty-seven PBDE (BDE-3, 7, 15, 17, 28, 47, 49, 66, 71, 77, 85, 99, 100, 119, 126, 138, 153, 154, 156, 183, 184, 191, 196, 197, 206, 207 209) were investigated by means of gas chromatography coupled to high resolution mass spectrometry (GC-HRMS). Specifically, the quantification was carried out by the isotopic dilution technique on a Trace GC Ultra gas chromatograph (Thermo Fisher Scientific, Milan, Italy) coupled to a high-resolution mass spectrometer (DFS, Thermo Fisher Scientific, Bremen, Germany). A full description of the instrumental parameters can be found in the SI.

#### QA/QC

To avoid contamination all material was cleaned (3X) with the following solvents of decreasing polarity: acetone, dichloromethane and n-hexane. Particular care was taken to minimize exposure to UV light during the whole analytical procedure. A procedural blank was included within each batch of six samples. The isotopic dilution technique was used for quantification according to the following criteria: (a) ratio between the two monitored ions within ± 15% of the theoretical value, and (b) limits of quantification (LOQs) corresponding to S/N of 10. Final concentrations were blank corrected and intrinsically corrected by recoveries. Satisfactory results were obtained from the analyses (n = 3) of the certified standard reference material SRM 1945 (“Organics in Whale Blubber”, NIST). Additional data related to QA/QC is provided in the Supplementary Information 2. Toxic Equivalent Quantities (TEQ) for dl-PCBs were determined using the World Health Organization (WHO)-2005 Toxic Equivalency Factors (TEF) for mammals^51^.

Non-detected values were calculated according to the medium bound with the substitution of non-detected compounds with the half of the detection limit (LOD).

### Statistical analyses

Statistical analyses were conducted using the software RStudio v.1.1.453 and graphic elaborations were realized using ggplot2 package^93^. Shapiro-Wilks test revealed non-normal distribution for PCBs and PBDEs, while a normal distribution was confirmed for CYP1A e CYP2B. Independent Mann–Whitney U tests were used to compare levels of major contaminant and cytochrome activity among different age groups. If a significant difference was obtained (*p* value < 0.05), post-hoc Dunn's multiple comparison was performed for individual comparisons of age groups. A regression analysis was used to evaluate the influence of PCBs and PBDEs on the expression of CYP1A1 and/or CYP2B. Spearman's rank correlation was adopted to measure the correlation between variables. All statistical analyses were considered significant at *p* < 0.05.

## Supplementary Information

Supplementary Tables.

Supplementary Information.
