# Supplementary material for: First assessment of POPs and cytochrome P450 expression in Cuvier’s beaked whales (Ziphius cavirostris) skin biopsies from the Mediterranean Sea
Source: Sci Rep. 2020 Dec 14;10:21891. doi: 10.1038/s41598-020-78962-3 (PMC7736872; doi:10.1038/s41598-020-78962-3)
Supplement: Supplementary file 1 — Supplementary Tables. [file 41598_2020_78962_MOESM1_ESM.docx]

**Supplementary Information 1**

**First assessment of POPs and Cytochrome P450 expression in Cuvier’s beaked whales (*Ziphius cavirostris*) skin biopsies from the Mediterranean Sea**

*Matteo Baini, Cristina Panti, Maria Cristina Fossi, Paola Tepsich, Begoña Jiménez, Frazer Coomber, Alice Bartalini, Juan Muñoz-Arnanz, Aurelie Moulins, Massimiliano Rosso*

**Table S1-1.** ΣPCBs, ΣICES7, and ∑PBDE concentration in the blubber of each sampled Cuvier’s beaked whale expressed in mg/kg lipid weight. Total TEQ is expressed in pg/g lipid weight. Cytochrome P450 1A and 2B isoform activity expressed in pmolCYP/mgprot.

| Sample | Age class | Sex | ∑PCBs | ΣICES7 | ∑PBDEs | TEQ | CYP1A | CYP2B |
| --- | --- | --- | --- | --- | --- | --- | --- | --- |
| ZCS1_1 | juvenile | M | 5.44 | 4.10 | 0.13 | 44.87 | 133.71 | 94.03 |
| ZCS1_2 | juvenile | M | 12.19 | 9.02 | 0.31 | 143.26 | 104.84 | 74.45 |
| ZCS1_3 | adult | M | 53.86 | 40.20 | 0.71 | 222.70 | 134.95 | 85.90 |
| ZCS1_4 | adult | F | 3.06 | 2.40 | 0.05 | 36.13 | 118.75 | 71.89 |
| ZCS1_5 | adult | M | 30.09 | 22.40 | 0.55 | 155.20 | 89.08 | 78.18 |
| ZCS1_6 | adult | M | 27.62 | 19.94 | 0.52 | 179.82 | 119.72 | 62.35 |
| ZCS1_7 | adult | M | 20.06 | 15.00 | 0.48 | 191.74 | 130.16 | 58.71 |
| ZCS2_1 | subadult | M | - | - | - | - | 137.76 | 130.88 |
| ZCS2_2 | adult | M | - | - | - | - | 143.07 | 123.74 |
| ZCS2_3 | subadult | M | 5.10 | 3.80 | 0.17 | 61.85 | 160.28 | 86.63 |
| ZCS2_4 | subadult | M | 17.42 | 12.95 | 0.33 | 153.34 | 134.14 | 89.10 |
| ZCS2_5 | adult | M | 10.73 | 8.05 | 0.25 | 88.05 | 191.66 | 111.73 |
| ZCS2_6 | subadult | M | 15.40 | 11.63 | 0.38 | 101.50 | 141.33 | 74.88 |
| ZCS2_7 | juvenile | M | 16.69 | 12.31 | 0.38 | 129.59 | 116.18 | 39.12 |
| ZCS2_8 | subadult | M | 45.21 | 32.61 | 1.01 | 241.50 | 210.20 | 135.77 |
| ZCS2_9 | subadult | M | 60.61 | 43.64 | 1.93 | 982.59 | 157.67 | 64.55 |
| ZCS2_10 | subadult | M | 9.64 | 7.22 | 0.23 | 104.96 | 232.42 | 132.16 |
| ZCS2_11 | adult | M | 26.64 | 18.80 | 0.45 | 107.12 | 209.13 | 101.68 |
| ZCS2_12 | subadult | M | 36.28 | 26.50 | 0.67 | 209.15 | 204.38 | 84.10 |
| ZCS2_13 | adult | F | 8.22 | 5.76 | 0.23 | 288.65 | 116.48 | 55.04 |
| ZCS2_14 | subadult | M | 11.25 | 8.36 | 0.27 | 85.84 | 156.00 | 59.46 |
| ZCS2_15 | juvenile | F | 12.97 | 9.84 | 0.23 | 92.19 | - | - |

**Table S1-2.** Mean, median, standard deviation, the minimum and maximum range of ΣPCBs, ΣICES7, TriCB, TetraCB, PentaCB, HexaCB, HeptaCB, ndl-PCB, dl-PCB, non-ortho PCB, mono-ortho PCB, Structure Activity Groups (SAG) 1-2-3-4, ∑PBDE, Penta-BDE mixtures, Octa-BDE mixtures, Deca-BDE mixtures in the blubber of Cuvier’s beaked whale of juvenile (n=4), subadult male (n=8), adult male (n=6) concentrations are expressed in mg/kg l.w. Total TEQ is expressed in pg/g lipid weight. Mean, median, standard deviation, the minimum and maximum range of BDE-47/PCB-153, BDE-47/BDE-99, BDE-99/BDE-100, BDE-153/BDE-154 ratio.

|  | **Juvenile (4)** | | | | | **Subadult (8)** | | | | | **Adult_male (6)** | | | | | **Adult_female (2)** | | | | |
| --- | --- | --- | --- | --- | --- | --- | --- | --- | --- | --- | --- | --- | --- | --- | --- | --- | --- | --- | --- | --- |
|  | **Mean** | **Median** | **SD** | **Min** | **Max** | **Mean** | **Median** | **SD** | **Min** | **Max** | **Mean** | **Median** | **SD** | **Min** | **Max** | **Mean** | **Median** | **SD** | **Min** | **Max** |
| **∑PCBs** | 11.82 | 12.58 | 4.68 | 5.44 | 16.69 | 25.11 | 16.41 | 19.91 | 5.10 | 60.61 | 28.17 | 27.13 | 14.40 | 10.73 | 53.86 | 5.64 | 5.64 | 3.65 | 3.06 | 8.22 |
| **TriCB** | 0.02 | 0.02 | 0.00 | 0.01 | 0.02 | 0.01 | 0.01 | 0.01 | 0.01 | 0.03 | 0.01 | 0.01 | 0.01 | 0.01 | 0.03 | 0.01 | 0.01 | 0.00 | 0.00 | 0.01 |
| **TetraCB** | 0.11 | 0.11 | 0.05 | 0.06 | 0.17 | 0.28 | 0.16 | 0.22 | 0.06 | 0.58 | 0.30 | 0.27 | 0.13 | 0.13 | 0.52 | 0.08 | 0.08 | 0.09 | 0.02 | 0.14 |
| **PentaCB** | 1.37 | 1.43 | 0.60 | 0.66 | 1.98 | 2.87 | 1.77 | 2.24 | 0.72 | 6.98 | 2.96 | 2.97 | 1.18 | 1.33 | 4.93 | 0.61 | 0.61 | 0.52 | 0.25 | 0.98 |
| **HexaCB** | 7.35 | 7.61 | 3.05 | 3.39 | 10.79 | 16.25 | 10.76 | 12.87 | 3.06 | 38.51 | 18.23 | 16.89 | 10.12 | 6.68 | 36.52 | 3.49 | 3.49 | 2.80 | 1.51 | 5.46 |
| **HeptaCB** | 2.97 | 3.39 | 1.15 | 1.32 | 3.77 | 5.70 | 3.70 | 4.63 | 1.25 | 14.52 | 6.67 | 6.36 | 3.20 | 2.57 | 11.87 | 1.46 | 1.46 | 0.24 | 1.28 | 1.63 |
| **ndl-PCB congeners** | 7.65 | 7.89 | 3.15 | 3.57 | 11.26 | 16.93 | 11.26 | 13.29 | 3.24 | 39.87 | 18.85 | 17.40 | 10.23 | 7.07 | 37.30 | 3.61 | 3.61 | 2.94 | 1.54 | 5.69 |
| **dl-PCB** | 1.23 | 1.30 | 0.50 | 0.56 | 1.74 | 2.54 | 1.48 | 2.08 | 0.62 | 6.38 | 2.70 | 2.60 | 1.20 | 1.11 | 4.80 | 0.59 | 0.59 | 0.47 | 0.26 | 0.92 |
| **non-ortho PCB** | 0.00 | 0.00 | 0.00 | 0.00 | 0.01 | 0.00 | 0.00 | 0.00 | 0.00 | 0.01 | 0.00 | 0.00 | 0.00 | 0.00 | 0.01 | 0.01 | 0.01 | 0.02 | 0.00 | 0.02 |
| **mono-ortho PCB** | 1.22 | 1.30 | 0.50 | 0.56 | 1.74 | 2.53 | 1.48 | 2.08 | 0.62 | 6.37 | 2.70 | 2.60 | 1.20 | 1.11 | 4.80 | 0.58 | 0.58 | 0.45 | 0.26 | 0.89 |
| **SAG-1** | 7.44 | 8.02 | 2.95 | 3.41 | 10.31 | 15.35 | 10.46 | 11.93 | 3.08 | 36.54 | 17.65 | 16.55 | 9.58 | 6.66 | 35.04 | 3.44 | 3.44 | 1.80 | 2.16 | 4.71 |
| **SAG-2** | 2.53 | 2.62 | 1.07 | 1.14 | 3.73 | 5.87 | 3.59 | 4.90 | 1.05 | 14.47 | 6.54 | 6.70 | 3.32 | 2.29 | 12.13 | 1.33 | 1.33 | 1.14 | 0.52 | 2.14 |
| **SAG-3** | 1.04 | 1.09 | 0.44 | 0.49 | 1.50 | 2.20 | 1.29 | 1.79 | 0.52 | 5.42 | 2.35 | 2.27 | 1.03 | 0.97 | 4.16 | 0.51 | 0.51 | 0.42 | 0.21 | 0.80 |
| **SAG-4** | 0.61 | 0.62 | 0.28 | 0.32 | 0.89 | 1.35 | 0.86 | 1.01 | 0.33 | 3.22 | 1.26 | 1.31 | 0.42 | 0.65 | 1.87 | 0.28 | 0.28 | 0.24 | 0.11 | 0.44 |
| **ΣICES7** | 8.82 | 9.43 | 3.44 | 4.10 | 12.31 | 18.34 | 12.29 | 14.24 | 3.80 | 43.64 | 20.73 | 19.37 | 10.77 | 8.05 | 40.20 | 4.08 | 4.08 | 2.38 | 2.40 | 5.76 |
| **Total TEQ** | 102.48 | 110.89 | 44.06 | 44.87 | 143.26 | 242.59 | 129.15 | 305.37 | 61.85 | 982.59 | 157.44 | 167.51 | 28.04 | 88.05 | 222.70 | 162.39 | 162.39 | 178.56 | 36.13 | 288.65 |
| **∑PBDEs** | 0.26 | 0.27 | 0.11 | 0.13 | 0.38 | 0.62 | 0.35 | 0.60 | 0.17 | 1.93 | 0.49 | 0.50 | 0.15 | 0.25 | 0.71 | 0.14 | 0.14 | 0.13 | 0.06 | 0.23 |
| **Penta-BDE mixture** | 0.24 | 0.25 | 0.10 | 0.12 | 0.35 | 0.59 | 0.33 | 0.55 | 0.16 | 1.78 | 0.48 | 0.48 | 0.15 | 0.24 | 0.68 | 0.13 | 0.13 | 0.11 | 0.05 | 0.21 |
| **Octa-BDE mixture** | 0.06 | 0.06 | 0.02 | 0.03 | 0.07 | 0.12 | 0.07 | 0.12 | 0.04 | 0.39 | 0.09 | 0.09 | 0.03 | 0.05 | 0.13 | 0.03 | 0.03 | 0.01 | 0.02 | 0.03 |
| **Deca-BDE mixture** | 0.01 | 0.01 | 0.01 | 0.00 | 0.02 | 0.00 | 0.00 | 0.00 | 0.00 | 0.01 | 0.00 | 0.00 | 0.00 | 0.00 | 0.00 | 0.01 | 0.01 | 0.01 | 0.00 | 0.01 |
| **BDE-47/PCB-153** | 0.03 | 0.03 | 0.01 | 0.02 | 0.03 | 0.03 | 0.03 | 0.01 | 0.02 | 0.04 | 0.02 | 0.03 | 0.01 | 0.01 | 0.03 | 0.03 | 0.03 | 0.02 | 0.01 | 0.04 |
| **BDE-47/BDE-99** | 2.36 | 2.59 | 0.52 | 1.59 | 2.68 | 2.60 | 2.63 | 0.14 | 2.28 | 2.73 | 2.62 | 2.58 | 0.16 | 2.44 | 2.90 | 2.22 | 2.22 | 1.00 | 1.51 | 2.93 |
| **BDE-99/BDE-100** | 1.37 | 1.31 | 0.17 | 1.25 | 1.63 | 1.30 | 1.28 | 0.15 | 1.10 | 1.57 | 1.57 | 1.55 | 0.20 | 1.36 | 1.86 | 1.49 | 1.49 | 0.19 | 1.36 | 1.63 |
| **BDE-153/BDE-154** | 0.38 | 0.37 | 0.05 | 0.33 | 0.46 | 0.36 | 0.35 | 0.04 | 0.31 | 0.45 | 0.45 | 0.45 | 0.06 | 0.36 | 0.52 | 0.40 | 0.40 | 0.11 | 0.32 | 0.48 |
