# Supplementary material for: First assessment of POPs and cytochrome P450 expression in Cuvier’s beaked whales (Ziphius cavirostris) skin biopsies from the Mediterranean Sea
Source: Sci Rep. 2020 Dec 14;10:21891. doi: 10.1038/s41598-020-78962-3 (PMC7736872; doi:10.1038/s41598-020-78962-3)
Supplement: Supplementary file 2 — Supplementary Information. [file 41598_2020_78962_MOESM2_ESM.docx]

**Supplementary Information 2**

**First assessment of POPs and Cytochrome P450 expression in Cuvier’s beaked whales (*Ziphius cavirostris*) skin biopsies from the Mediterranean Sea**

*Matteo Baini, Cristina Panti, Maria Cristina Fossi, Paola Tepsich, Begoña Jiménez, Frazer Coomber, Alice Bartalini,* *Juan Muñoz-Arnanz, Aurelie Moulins, Massimiliano Rosso*

**Supplementary Information on contaminant analysis**

1. **Reagents and standards**

All solvents used in this work were of high purity (pesticide residue grade or equivalent). Dichloromethane, n-hexane, and toluene were purchased from J.T. Baker (Deventer, The Netherlands). LGC Standards GmbH (Wesel, Germany) was the provider for n-nonane. Anhydrous sodium sulfate was purchased from J.T. Baker (Deventer, The Netherlands). The analytical standards used in this study are summarized in table S2-1.

**Table S2-1**. Labeled and native chemical standards used in this study.

| **Group** | **Congeners / Isomers** | **Supplier** |
| --- | --- | --- |
| native PCBs | PCB-28, -52, -77, -81, -101, -105, -114, -118, -123, -126, -138, -153, -156, -157, -167, -169, -180, -189 (calibration solution **WM48-CVS**) | Wellington Laboratories  (Guelph, Ontario, Canada) |
| labeled PCBs | ^13^C_12_-PCB-28, -52, -77, -81, -101, -105, -114, -118, -123, -126, -138, -153, -156, -157, -167, -169, -180, -189 (calibration solution **WM48-CVS** and PCB mixes **P48-W-ES** and **P48-M-ES**) |  |
|  | ^13^C_12_-PCB-70, -111, -170 (PCB mix **P48-RS**) |  |
| native PBDEs | BDE-3, -7,- 15, -17, -28, -47, -49, -66, -71, -77, -85, -99, -100, -119, -126, -138,- 153, -154, -156, -183, -184, -191, -196,-197,-206, -207, -209 (calibration solution **BDE-CVS-G)** |  |
| labeled PBDEs | ^13^C_12_-BDE-3, -15, -28, -47, -99, -100, -126, -153, -154, -183, -197, -207, -209 **(**calibration solution **BDE-CVS-G** and mix **MBDE-MXG)** |  |
|  | ^13^C_12_-BDE-79, -138, -206, **(**mix **MBDE-ISS-G)** |  |

1. **Sample extraction and purification with the DEXTech+ system**

Fresh blubber samples (from 0.0507 to 0.3736g, Table S2-2) were homogenized with 15g of anhydrous sodium sulfate (Na_2_SO_4_) and spiked with a suite of ^13^C-labeled standards:

**Table S2-2.** Amount (pg) used surrogate standards spiked prior to extraction.

| ^13^C_12_-PCBs (**mixes P48-W-ES, P48-M-ES**) | ^13^C_12_-PBDEs (**mix MBDE-MXG**) |
| --- | --- |
| 200 pg for all congeners | 1000 pg for mono- to penta-brominated congeners  2000 pg for hexa- to octa- brominated congeners  5000 pg for nona- to deca-brominated congeners |

Following a proper equilibrium time after surrogate addition, samples were Soxhlet extracted for 24h with ̴100mL of n-hexane: DCM (9:1) mixture. Extracts were concentrated by rota-evaporation and purified by using the automated sample preparation system DEXTech+ (LCTech GmbH, Dorfen, Germany). Final extracts of 10 mL including 500 µL of acetone as solvent modifier were introduced in the DEXTech+ sytem, operated in its alumina configuration with three columns: 1) acidic silica gel, 2) aluminum oxide and 3) carbon, and three different solvents: 1) n-hexane, 2) n-hexane:dichloromethane (1:1) and 3) toluene. This 3-column set up rendered two fractions: F1 containing all PCBs except the four non*-ortho* congeners and all PBDEs, and F2 containing the non-*ortho* PCB congeners. SMART silica gel columns, designed for samples with up to 1.5 g of fat, were used. Each run lasted for 45 min with a total solvent consumption of 205mL (collected volume of 24 mL of n-hexane:DCM for F1 and 10 mL of toluene for F2). Final extracts were evaporated using a TurboVap® (Zymarck Inc., Hopkinton, MA, USA) system until ̴1 mL, transferred to vials, and dried under a gentle stream of nitrogen. Fractions were reconstituted in ^13^C-labeled injection standards: 20 µL (F1) of PCBs (mix P48-RS) + PBDEs (mix MBDE-ISS-G) and 10 µL (F2) of PCBs (mix P48-RS) prior to instrumental analysis.

1. **Lipid determination**

The lipid content of each sample was determined gravimetrically. Each extract was first rota-evaporated close to dryness. Afterwards, they were transfer to an oven for 30 min at 105°C. They were kept covered overnight and weighed the following day. Weight differences for each flask before and after extraction were assumed as lipid content. Lipid content for each sample is shown in Table S2-3.

**Table S2-3.** Wet weight (w.w.) and lipid weight (l.w.) of the analysed samples

| Sample ID | **w.w.**  **(g)** | **l.w.**  **(g)** | **l.w.**  **(%)** |
| --- | --- | --- | --- |
| S1_1 | 0.1423 | 0.078 | 54.8 |
| S1_2 | 0.0647 | 0.007 | 10.8 |
| S1_3 | 0.1273 | 0.022 | 17.3 |
| S1_4 | 0.3037 | 0.078 | 25.7 |
| S1_5 | 0.1325 | 0.020 | 15.1 |
| S1_6 | 0.1459 | 0.026 | 17.8 |
| S1_7 | 0.0778 | 0.014 | 18.0 |
| S2_3 | 0.2430 | 0.083 | 34.2 |
| S2_4 | 0.3163 | 0.116 | 36.7 |
| S2_5 | 0.1914 | 0.049 | 25.6 |
| S2_6 | 0.3237 | 0.085 | 26.3 |
| S2_7 | 0.1541 | 0.032 | 20.8 |
| S2_8 | 0.1216 | 0.010 | 8.2 |
| S2_9 | 0.0517 | 0.001 | 1.9 |
| S2_10 | 0.0972 | 0.028 | 28.8 |
| S2_11 | 0.3736 | 0.122 | 32.7 |
| S2_12 | 0.0733 | 0.007 | 9.5 |
| S2_13 | 0.0507 | 0.016 | 31.6 |
| S2_14 | 0.1453 | 0.052 | 35.8 |
| S2_15 | 0.0724 | 0.017 | 23.5 |

1. **Instrumental determination**

Sixteen PCBs (#28, 52, 77, 81, 101, 105, 114, 118, 123, 126, 153, 156, 157, 167, 169, 180, 189) and twenty-seven PBDEs (# 3, 7, 15, 17, 28, 47, 49, 66, 71, 77, 85, 99, 100, 119, 126, 138, 153, 154, 156, 183, 184, 191, 196, 197, 206, 207, 209) were quantified by GC-HRMS on a Trace GC Ultra gas chromatograph (Thermo Fisher Scientific, Milan, Italy) coupled to a high-resolution mass spectrometer (DFS™, Thermo Fisher Scientific, Bremen, Germany). One μL of each extract was injected in splitless mode at a temperature of 260°C using helium as carrier gas at a constant flow mode. GC separation of PBDEs was achieved using a 15 m × 0.25 mm × 0.10 μm Rxi-5Sil MS column (Restek, USA). GC separation of PCBs was achieved using a 60 m × 0.25 mm × 0.25 μm DB-5MS column (Agilent J&W, USA). The different oven temperature programs and carrier flows used for both families of target compounds are shown in Table S2-4. Positive electron ionization (EI+) was used operating in selected ion monitoring (SIM) mode at a 10,000 resolving power.

**Table S2-4**. Oven temperature programs and carrier flow values used in the analysis of the target contaminants.

| **PCBs** | | | **PBDEs** | | |
| --- | --- | --- | --- | --- | --- |
| ramp (°C/min) | T (°C) | hold time  (min) | ramp (°C/min) | T (°C) | hold time  (min) |
|  | 140 | 1 |  | 130 | 4.2 |
| 20 | 200 | 3 | 20 | 200 | 3 |
| 3 | 275 | - | 3 | 310 | 8 |
| 30 | 310 | 10 |  |  |  |
| helium flow 1.3 mL/min | | | helium flow 1.5 mL/min | | |

1. **QA/QC**

Quantification was based on the isotopic dilution technique with the following criteria: (a) ratio between the two monitored ions within ±15% of the theoretical value, and (b) limits of quantification (LOQs) corresponding to S/N of 10. Linear calibration curves for PCBs (6 points from 0.1 to 1000 pg/µL, calibration solutions WM48-CVS) and PBDEs (5 points from 1.0 to 2000 pg/µL, calibration solutions BDE-CVS-G) were daily checked. When quantifiable levels of a given analyte were found in a procedural blank, they were subtracted from the batch of samples associated to that blank. Average recoveries for the used surrogates and average LOD values (calculated as 3 times the relationship S/N) are summarized in Table S2-5.

**Table S2-5.** Recovery values of labeled surrogates and average LODs for target compounds.

| **Compound** | **Recovery**  **(%) ± SD** | **Average LOD**  **(pg/g l.w.)** | **Compound** | **Recovery**  **(%) ± SD** | **Average LOD**  **(pg/g l.w.)** |
| --- | --- | --- | --- | --- | --- |
| **PCBs**  PCB-28  **^13^C_12_-PCB-28**  PCB-52  **^13^C_12_-PCB-52**  PCB-77  **^13^C_12_-PCB-77**  PCB-81  **^13^C_12_-PCB-81**  PCB-101  **^13^C_12_-PCB-101**  PCB-105  **^13^C_12_-PCB-105**  PCB-114  **^13^C_12_-PCB-114**  PCB-118  **^13^C_12_-PCB-118**  PCB-123  **^13^C_12_-PCB-123**  PCB-126  **^13^C_12_-PCB-126**  PCB-138  **^13^C_12_-PCB-138**  PCB-153  **^13^C_12_-PCB-153**  PCB-156  **^13^C_12_-PCB-156**  PCB-157  **^13^C_12_-PCB-157**  PCB-167  **^13^C_12_-PCB-167**  PCB-169  **^13^C_12_-PCB-169**  PCB-180  **^13^C_12_-PCB-180**  PCB-189  **^13^C_12_-PCB-189** | 74.4± 11.3  81.4± 11.0  78.3± 9.0  79.9± 9.2  85.3± 8.8  88.1± 10.7  86.4± 8.1  83.2± 9.1  89.0± 10.1  88.7± 9.7  104± 14  91.6± 11.2  86.0± 9.3  93.3± 9.6  82.9 ± 9.9  94.2 ± 9.1  91.5 ± 9.4  86.3 ± 8.1 | 52.0  121  16.8  15.7  197  165  173  185  170  34  104  82.6  95.9  90.5  98.7  15.9  67.7  56.0 | **PBDEs**  BDE-3  **^13^C_12_-BDE-3**  BDE-7  BDE-15  **^13^C_12_-BDE-15**  BDE-17  BDE-28  **^13^C_12_-BDE-28**  BDE-47  **^13^C_12_-BDE-47**  BDE-49  BDE-66  BDE-71  BDE-77  BDE-85  BDE-99  **^13^C_12_-BDE-99**  BDE-100  **^13^C_12_-BDE-100**  BDE-119  BDE-126  **^13^C_12_-BDE-126**  BDE-138  BDE-153  **^13^C_12_-BDE-153**  BDE-154  **^13^C_12_-BDE-154**  BDE-156  BDE-183  **^13^C_12_-BDE-183**  BDE-184  BDE-191  BDE-196  BDE-197  **^13^C_12_-BDE-197**  BDE-206  BDE-207  **^13^C_12_-BDE-207**  BDE-209  **^13^C_12_-BDE-209** | 58.0 ± 12.4  71.7 ± 10.7  78.5 ± 13.9  94.5 ± 9.8  89.9 ± 14.5  84.8± 13.7  83.1± 11.0  94.5± 14.1  87.0± 14.5  91.9± 13.8  87.1± 22.4  103.9± 18.3  66.4± 14.1 | ND  ND  ND  103  98.4  65.1  96.0  104  92.4  61.2  141  111  123  101  ND  126  107  101  132  57.1  52.0  51.1  28.9  29.7  57.8  48.8  210 |

ND: stands for non-detected.

The accuracy of the analytical method was assessed by analyzing a triplicate of the certified standard material SRM 1945 (‘Organics in Whale Blubber’, NIST). Satisfactory results were obtained for all target compounds and they can be found at (Bartalini et al., 2019). The precision for the quantification method was checked by reanalyzing three different blubber samples in three different days within two weeks obtaining RSDs lower than 14% for all target analytes.

**6. Congeners included in the different groupings of PCB:**

TriCB: CB-28

TetraCB: CB-52, 77, 81

PentaCB: CB-101, 105, 114, 118, 123, 126

HexaCB: CB-138, 153, 156, 157, 167, 169

HeptaCB: CB-180, 189

ndl-PCB congeners: CB-28, 52, 101, 138, 153, 180

dl-PCB: CB-77, 81, 105, 114, 118, 123, 126, 156, 157, 167, 169, 189

non-ortho PCB: CB-77, 81, 126, 169

mono-ortho PCB: CB-105, 114, 118, 123, 156, 157, 167, 189

ICES 7: CB-28, 52, 101, 118, 153, 138, 180

Structure Activity Groups (SAG) 1: CB-153, 180

SAG 2: CB-153, 180

SAG 3: CB-138

SAG 4: CB-52, 101

**7. Congeners included in the different mixtures of PBDE:**

Penta-BDE mixture: BDE-47, 99, 100, 153, 154

Octa-BDE mixture: BDE-153, 183

Deca-BDE mixture: BDE-209
